# A model to explain self-medication by Iranian people: a qualitative grounded theory study

**DOI:** 10.1186/s12889-019-7953-0

**Published:** 2019-12-02

**Authors:** Zhila Fereidouni, Majid Najafi Kalyani

**Affiliations:** 1 School of Nursing, Fasa University of Medical Sciences, Fasa, Iran; 2 School of Nursing and Midwifery, Shiraz University of Medical Sciences, Shiraz, Iran

**Keywords:** Grounded theory, Self-medication, Conceptual model, Iran

## Abstract

**Background:**

Self-medication (SM) is a common and global health problem. The process of attempting SM is still unclear. Exploration of SM and its contributing factors would help policymakers design and develop preventive programs. This qualitative study aimed to explore the process of attempting SM among Iranian people.

**Methods:**

This grounded theory (GT) study was conducted among people with the experience of attempting SM (*n* = 17) and medical staff (*n* = 9) in Iran selected via semi-structured interviews. The recorded and transcribed interviews were analyzed using open, axial, and selective coding based on Strauss and Corbin’s (1998) approach.

**Results:**

The study results revealed that people sought to deal as simply and quickly as possible with their illnesses/symptoms according to their attitudes towards and perceptions of illnesses/symptoms as well as their economic and social problems. This simple and quick approach was the participants’ main concern, resulting in taking decision-making strategies as SM facilitators. SM, in turn, provided short-term improvement and temporary satisfaction as a predominant outcome. Overall, “to avoid being trapped in the vortex of illness” was the central category, which covered and connected all the other categories developed in this study.

**Conclusions:**

The elements of this model could be used as a guide for healthcare policymakers to design preventive programs and to plan for increasing people’s knowledge about the complications and consequences of SM. In addition, identification of barriers to referral to physicians and treatment of illnesses through the right way as well as reducing the health system’s problems would help reduce SM.

## Background

SM is an activity practiced by ordinary people with the aim of dealing with their health problems without consulting healthcare practitioners [1]. In other words, SM refers to the use of over-the-counter drugs to treat a whole host of ailments without medical supervision [2]. Medication overuse in particular and SM in general are two major social, health, and economic problems in many countries, including Iran [3]. Research findings have indicated that a large percentage of Iranian patients practiced SM before seeking for care at public service [4–7].

SM may involve the use of herbal [8] or chemical drugs [9], access to over-the-counter drugs, consumption of the previously prescribed drugs in similar cases, shared use of the prescribed drugs for one person by family members and partners, consumption of excess drugs at home, drug misuse on friends’ and peers’ advice, and previous experience with medicine [10–14].

SM has led to the expansion of adverse effects, such as antibiotic and drug resistance [10], inefficient and prolonged treatment [15], harmful toxicity and implications [16], and drug dependence [15]. Despite the few positive effects of SM on improvement of health conditions [17], the patient is exposed to more adverse effects and greater drug resistance. This also results in disruption in the pharmaceutical market, waste of money, and increased per capita of drug use [18, 19].

Healthcare system in Iran consists of public and private sectors. The public sector plays important roles in primary, secondary, and tertiary health services. The private sector, on the other hand, provides secondary and tertiary healthcare services [20]. In Iran, pharmacies in public and private settings are allowed to sell drugs in accordance with physicians’ prescriptions or without prescription as over-the-counter drugs [21]. However, selling drugs by other stores is considered to be illegal [21].

Many Iranian researchers have thus far focused on the issue of SM. A quantitative study by Jafari et al. [22] revealed an 83% prevalence of SM among elderly individuals in Iran. A study on Iranian university students by Sararoudi et al. [23] also showed that 76.6% of the students had a history of SM with analgesic drugs. Similarly, the meta-analysis performed by Azami Aghdash et al. [3] was indicative of a 53% prevalence of SM in Iran. In spite of focusing on the issue of SM, none of these research works adopted a qualitative approach to SM.

Given the fact that SM is a multi-dimensional process and potentially has different implications in different cultures and societies, it is necessary to identify the influential factors on this process according to the social context and cultural aspects and values to find a solution by means of appropriate and realistic planning. A primary step, to begin with, is to collect a set of qualitative in-depth information from the SM practitioners themselves. Due to the mentioned research gap, the present qualitative study aims to explain the process of SM among Iranian people.

## Methods

In methodological terms, this qualitative study was conducted based on Strauss and Corbin’s version of GT (1998). The study aimed at investigating the SM process during 2014–2018. The main question of the study was “how is SM practiced by Iranians”. To this end, the qualitative research method based on the GT approach was taken into account as the most effective approach to the question under consideration [24]. GT is a methodological approach for qualitative researches that allows researchers to interpret a process and its components based on the data derived from the participants’ experiences [25].

The participants in this study were first selected from the individuals with a history of SM through purposive sampling. Theoretical sampling was applied after the development of classes to establish the relationships. The inclusion criteria of the study were having a recent experience of SM and being willing to share the experience. Initially, 11 participants (with a history of SM) were selected through purposive sampling. After the development of the classes, six other participants were interviewed to establish the interclass relationships and to extract the final theory. Apart from the participants with a history of SM, three pharmacists and six general practitioners and specialists were enrolled in the process of theoretical sampling to develop and establish the model themes and interclass relationships. The process continued to the point of data and theoretical saturation.

The data were collected via semi-structured face-to-face and online interviews. The location and time of the interviews were determined and then agreed by the participants. Every 25-40-min interview started with a general question, “*How do you describe your SM experience*?”. This was followed by a series of exploratory questions according to the participants’ responses to gain a full understanding of the SM process. Some of these questions were as follows: “could you please explain more about your decision?” and “could you please mention the basis of your decision for SM?” (see Additional file 1). These questions included an examination of facilitators, inhibitors, and theme-based factors affecting the process. In online interviews, the participants were asked to provide a detailed answer to a certain set of questions and, if necessary, more questions were added later. The face-to-face interviews were recorded with the participants’ written informed consent. Afterwards, the online and face-to-face interviews were all transcribed. In addition to the interviews, some reminders were used to collect information and complete the process and interclass relationships. These reminders revealed a primary analysis after the interviews with the participants and helped generate the final process of the study.

The constant comparative method based on Strauss and Corbin’s version of GT (1998) was employed in order to analyze the data extracted from the interviews. In doing so, the records were transcribed immediately after each interview. After reviewing, they were analyzed manually through open, axial, and selective coding to reach the point of theoretical saturation. In open coding, the interview scripts were read for several times and then, the main theme-based sentences were extracted and coded. These codes were divided into certain classes based on similarity. In axial coding, the classes were related to their subclasses and consequently, they were arranged around a common axis. Finally, the central theme was extracted in selective coding. Another method for collecting and analyzing the data was the use of reminders at all stages of data analysis to relate the classes and themes and develop a conceptual framework [25].

This study was approved by Fasa University of Medical Sciences (grant No. 93093). Indeed, the researchers observed all ethical considerations for a qualitative study, including explaining the study purpose and methodology, obtaining written informed consents, offering the possibility of resigning voluntarily at any time, and ensuring the participants’ right for privacy.

The criteria suggested by Lincoln and Guba were used to enhance the accuracy of the study results [25]. Long-term involvement of the researcher with the study process as well as continuous data examination aimed to increase data credibility. The process of data validation was conducted through reviews by both participants and supervisors. Other subjects who had a history of SM but did not participate in the study were provided with the results and were asked to compare them to their own experiences. Other approaches adopted in the study to enhance the accuracy of the results were the inclusion of participants of different ages and genders, application of different data collection methods, and close examination of the study process from data coding and analysis up to the final theory development by a group of three professors experienced in qualitative researches. The study findings were reported based on the consolidated criteria for reporting qualitative research (COREQ) checklist.

## Results

**Fig. 1:**
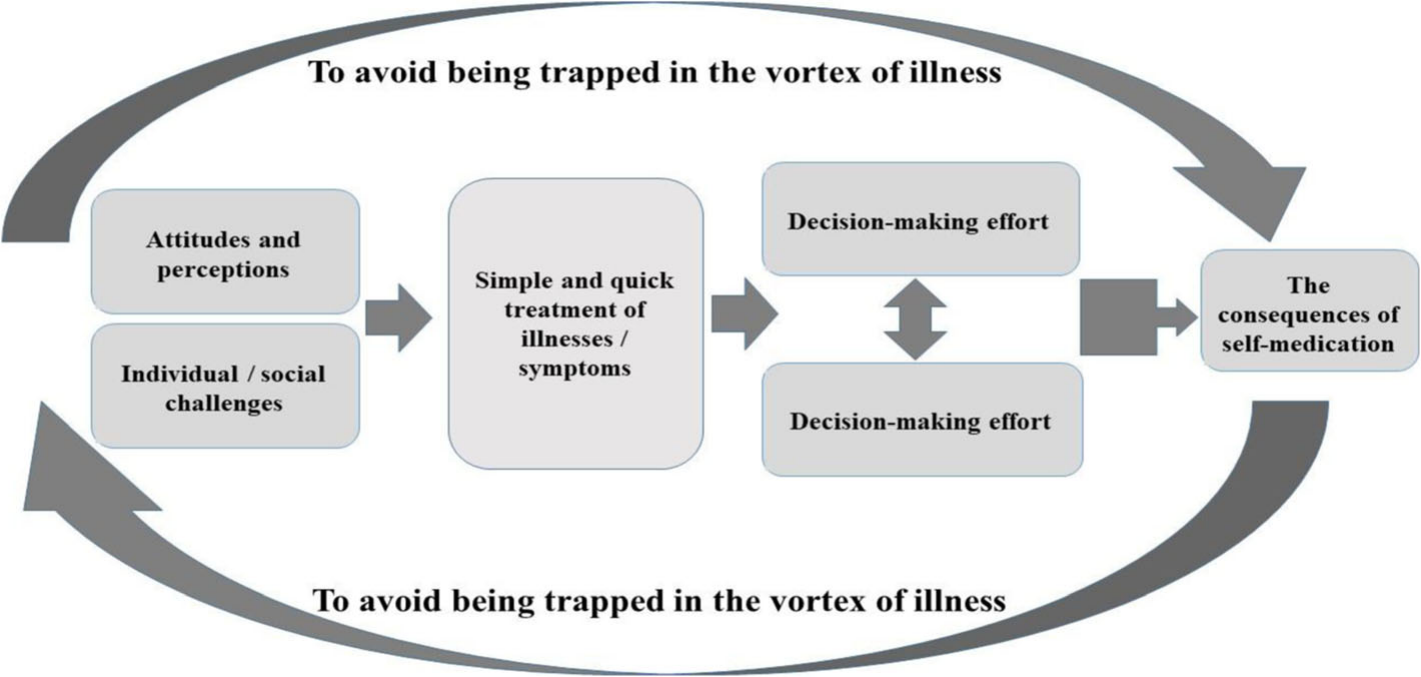
Conceptual model of the process of SM among Iranian people. People sought to deal as simply and quickly as possible with their illnesses/symptoms according to their attitudes towards and perceptions of the illnesses/symptoms as well as their economic and social problems. This simple and quick approach was the participants’ main concern, resulting in taking decision-making strategies as SM facilitators. SM, in turn, provided short-term improvement and temporary satisfaction. However, dissatisfaction was a consequence of SM. The central class; i.e., “to avoid being trapped in the vortex of illness”, covered and connected all other classes developed in this study

The study results, in the form of a conceptual model, revealed that people sought to deal as simply and quickly as possible with their illnesses/symptoms according to their attitudes towards and perceptions of the illnesses/symptoms as well as their economic and social problems. This simple and quick approach was the participants’ main concern, resulting in taking decision-making strategies as SM facilitators. SM, in turn, provided short-term improvement and temporary satisfaction. However, dissatisfaction was a consequence of SM. The central class; i.e., “to avoid being trapped in the vortex of illness”, covered and connected all other classes developed in this study (Fig. 1).

### Attitudes and perceptions

When facing the symptoms of an illness, people tend to consider their problem as unimportant and avoid taking it seriously. Individuals’ attitudes towards the health care system in the public and private sectors play a key role in the process of SM. This study also revealed that the participants’ attitudes towards and perceptions of diseases, SM, and physicians played a major role in SM. They suggested that the mildness of a disease was a compelling reason for SM.

“... When I have a cold, I say to myself that it is not serious and I can deal with it with a few pills. Why going to a doctor? It is simply treated with some pills ...” (a 51-year-old).

The people’s perceptions of temporary illnesses and symptoms significantly influenced their decision to practice SM. They believed that they could treat such temporary cases easily and quickly through SM.

“... Whenever I get headaches, I feel it is temporary and not important. So, I take medicines so that I don’t have to see a doctor ...” (a 53-year-old).

The SM practitioners regarded seeing a doctor as a total waste of time and believed that they were able to take the medications of the same treatment a doctor might prescribe after spending too much time and money.

“... I had a stomach problem. I did go to the doctor several times. After undergoing many tests and radiographical examination over almost 10 days, he prescribed ranitidine pills for my stomach pains. I was already aware of that. Since then, I merely take a pill whenever I feel stomach pains ...” (a 65-year-old).

According to the participants, the ineffective prescribed treatments significantly accounted for their SM behavior.

“... I was almost sure that going and not going to the doctor would have the same result, so I chose SM ...” (a 27-year-old).

Furthermore, most of the participants expressed their mistrust and negative attitudes towards physicians and medical interventions as two major factors facilitating their SM behavior. They also claimed that a large number of follow-ups, tests, and radiographical assessments were prescribed by physicians for financial purposes.

“... You feel that this (going to the doctor) is all about financial exploitation to prescribe many tests ... and that what lies behind the phrase “come back again a month later for another visit” is nothing but financial incentives ...” (a 51-year-old).

From the perspective of the physicians participating in this study, the mentioned mistrust and the resulting SM were attributed to the people’s avoidance of facing the possible need for advanced lengthy paraclinical procedures.

“... People are usually afraid of paraclinical examinations and costs, which may be one of the reasons why they don’t trust physicians ...” (a 37-year-old).

The participants with a history of SM stated that what largely encouraged them to practice SM were futile medical interventions and rare honesty among physicians.

“... I am afraid that the medical profession has today become more of a business than a profession and that honesty is rarely found among the recent generation of physicians. So, I prefer trying SM ...” (a 35-year-old).

Another negative attitude affecting the prevalence of SM was the widespread assumption that physicians worked in partnership with pharmacists.

“... Many patients believe that there is a financial relationship between physicians and nearby pharmacies due to which, drugs are mostly prescribed with financial incentives. Accordingly, they do prefer to go directly to the pharmacy for SM purposes ...” (a 36-year-old).

### Individual/social challenges

The high costs of diagnosis, treatment, and physician office visit on the one hand and tight family budgets on the other hand were the underlying factors encouraging SM. The inability to cover treatment costs, time-consuming medical procedures, and insurance problems were the major challenges in this regard. Most of the participants mentioned the costly treatments and their financial inability as what justified their SM behavior.

“... I was not able to pay the visit and other charges anymore ... So, I took drugs myself without seeing a doctor ...” (a 37-year-old).

Besides the costly visit charges, many participants suggested that the need to undergo lengthy treatment procedures was a big problem leading them to SM.

“... The physicians used to diagnose your illness very soon. But today’s physicians act differently, waste your time, and cannot treat you in one visit ...” (a 51-year-old).

According to the physicians participating in the study, the easy access to medications and the cheaper nature of SM encouraged people to practice SM.

“... The availability of cheap drugs plays a key role in people’s tendency towards SM ...” (a 37-year-old).

The participants also counted lengthy procedures, which consequently imposed financial burdens, as a facilitating factor influencing their decision to practice SM.

“... I am a daily laborer. If I don’t go to work one day, I will have no money to cover the daily costs of living. This means that if I go to the doctor for a small problem, this will take a whole day and I will have no money to feed my family ...” (a 45-year-old).

Lack of social support (e.g. insurance coverage) and costly visit charges had forced some participants to go for SM as the only solution.

“... My son has no insurance. Once he had a slight cold and went to the clinic, he had to spend 100-120 thousand tomans for the visit charge and medications. Therefore, I buy him the drugs whenever he has a cold. So, he is treated with a far lower cost ...” (a 53-year-old).

Inappropriate insurance coverage was another challenge faced by those practicing SM.

"... I enjoy no social security benefits. Then, I have no choice but going for SM due to financial problems to reduce the expenses ...” (a 50-year-old).

Another participant said:

“... I went to the doctor for my allergy and it costed me 200 thousand tomans even with my insurance, as it didn’t fall within the scope of the coverage. Thereafter, I buy and take my drugs personally so as not to have to pay the visit charges.” (a 35-year-old).

### Simple and quick treatment of illnesses/symptoms

The main concern of the present study participants was the simple and quick treatment of illnesses/symptoms in SM practices. According to all the participants, their SM behavior was aimed to achieve rapid improvements.

“... I had a terrible toothache. I took medicines and did not go to a dentist to relieve my pain as quickly as possible ...” (a 31-year-old).

The participants practiced SM to deal with their acute problems, which were reportedly resolved in most cases shortly after SM.

“... Once I had a severe headache and I was desperately in search of a solution. I opened the refrigerator and swallowed Ibuprofen. Then, I took a short rest and I felt well after an hour ...” (a 40-year-old).

The main reason behind the common SM behavior was the immediate treatment of ailments and the related symptoms. All the participants considered the expected immediate treatment and relief as the primary cause of SM.

“... As I sought for an immediate relief, I took medicine without seeking for a doctor. I said to myself why being in pain and wasting time to go to a doctor ...” (a 41-year-old).

Another participant mentioned:

“... When I get sick, e.g. a headache ..., my parents feel a pity and give me a glass of water and a pill, which I take for a relief ...” (a 35-year-old).

### Decision-making effort

The individuals who tended to practice SM used a wide range of strategies to deal with their main problems. These strategies included searching for information, consulting with others, and acting on their past SM experiences. After feeling an urgent need for treatment, the participants reportedly obtained information from friends, family members, peers, and non-medical professionals like pharmacists.

“... I had hair loss. As I was sure it was futile to go to the doctor, I used the information on the websites of reputable specialists for treatment purposes ...” (a 27-year-old).

In this context, searching for information via the Internet was one of the most common strategies, which allegedly facilitated the individuals’ decision-making process. Through an Internet search, according to the participants, they learned the treatment method and bought the required drugs from pharmacies.

“... I suffered from painful urination and fever about two months ago. As it was very difficult for me to reach the specialist by going first to the family physician and then to be referred to the specialist, I used the Internet and found out that I had urinary infection. I read some articles and then, I bought the necessary medications from the pharmacy ...” (a 66-year-old).

Information exchange in pharmacies was another strategy to make decisions and gain information about diseases and treatments. A large percentage of the participants believed that this was a very time/cost-efficient strategy.

“My kid used to have a skin allergy. I went to the doctor several times and ended without outcomes. One day, I went to the pharmacy and the seller advised me to use a certain ointment. I did accordingly and my kid exhibited improvements after a while ...” (a 36-year-old).

According to the pharmacists participating in this study, many people believe that the pharmacists know the medications far better than the physicians do. Without consulting a doctor, they thus go directly to the pharmacists to obtain the required medical information for SM purposes.

“... The patients prefer to consult pharmacists rather than physicians since they believe that the pharmacists have a deeper knowledge of drugs and medications ...” (a 35-year-old).

The SM practitioners also shared their personal experiences, which further facilitated the process.

“... I had a terrible toothache. My brother said that once he had such a pain and used phenylephrine. It had perfectly worked for him. So, I did accordingly and achieved the same result ...” (a 51-year-old).

The influence of family members and their advice was another key factor in SM among the participants. Most of the participants found their family members’ agreement on SM to be a compelling reason.

“... I had a stomachache on several occasions. My daughter and my son, who are nurses, recommended using a certain medicine. I did so and got well. Since then, I have not been in hospitals or doctor offices ...” (a 63-year-old).

The past experiences and knowledge of illnesses/symptoms and treatments were reported as another facilitator of SM. The participants believed that their previous knowledge of their illnesses or drugs was an inhibiting factor preventing them from seeing a doctor while encouraging them to practice SM.

“... As I had already suffered from this illness and I was aware of the treatment method, I directly went to the pharmacy without consulting a doctor. Then, I used the medicines ...” (a 27-year-old).

### The consequences of SM

SM has a range of consequences. Temporary satisfaction was identified as the main consequence from the participants’ perspective. The components of SM satisfaction included problem-solving, cost-efficiency, and time-efficiency. Many participants referred to the relieved symptoms as the outcome of the SM process. They also believed that this relief brought them temporary satisfaction.

“... I have had a stomach disorder for twenty years. I take a ranitidine pill every night to sleep comfortably. I feel satisfied as it relieves my pain ...” (a 51-year-old).

SM temporarily resolved the individuals’ problems and resulted in a short-term satisfaction. However, it would ultimately force patients to see a doctor and pay the visit charge in the long run.

“... I once had a headache when my family members recommended going for SM. Although I was feeling well for a while, my headache became very severe later. I was worried that there might be a brain tumor. Then, I had to go to the doctor who prescribed electroencephalography. Finally, it turned out to be a nervous condition ...” (a 35-year-old).

A history of taking similar medications or suffering from similar illnesses reportedly played an influential role in the effectiveness of and satisfaction with SM.

“... I usually wait for a while when I have a headache. Then, I take a medicine if I do not get well. Whenever I do so, I feel well soon ...” (a 40-year-old).

In addition to symptoms relief, another consequence of SM was evidently satisfaction with cost-efficiency. The individuals practicing SM were often satisfied with their behavior owing to the resultant cost savings and easy access to medications.

“... You do not need to see a doctor and pay the related charges for a small problem like a headache, which is a very good point. Accordingly, I easily and quickly obtain the necessary drug at a lower cost. Besides, it has the same outcome as going to the doctor ...” (A 44-year-old).

Satisfaction with time-efficiency was another component of satisfaction with SM among the practitioners.

“... A minor problem like headache takes half a day to improve. There is no waste of time whenever I take a pill according to my past experience. I have always been satisfied with what I have done ...” (a 51-year-old).

Quite the contrary, another consequence of SM was reportedly the long-term dissatisfaction with SM. According to some of the SM practitioners, the sense of dissatisfaction was attributed to the prolonged costly treatment course as the result of deliberate avoidance of standard treatments and, thus, the delayed effective treatment.

“... When I was suffering from a severe pain, I took medicines and felt better for a while. After a few days, I felt the same and I had to pay a lot of money for treatment ...” (a 65-year-old).

In addition to cost-inefficiency, the patients mentioned the prolonged treatment course as one of the consequences of SM.

“... If I had gone to the hospital on the first day I felt sick and I had not acted on non-professional advices, this foot pain would have got well after a month without a big waste of money and time ...” (a 44-year-old).

## Discussion

This GT study aimed to explain the process of SM occurring among Iranian people. The study results indicated that the main stimulant of the SM process was people’s willingness to deal as simply and quickly as possible with illnesses/symptoms. Not only this process occurred based on people’s attitudes towards their illnesses and physicians, but it was also affected by economic and social problems. The process also involved strategies, such as seeking for information and consulting others about their related experiences, which contributed to the prevalence of SM. Moreover, the consequences of SM ranged from satisfaction to dissatisfaction, in which the resulting temporary satisfaction and relief from illnesses/symptoms was the dominant dimension. Furthermore, “*To avoid being trapped in the vortex of illness*” was the central category in this study, thereby connecting all the other categories. The components of this conceptual model are going to be discussed in details in the following paragraphs.

According to the study results, the patients’ attitudes toward illnesses and physicians influenced their practice of SM. The individuals who practiced SM justified their behavior by not taking their illnesses seriously and believing that it was both minor and transient. In this regard, Loyola Filho et al. [13] suggested that the above-mentioned approach played a central role in SM. Beza et al. [12] and Jafari et al. [22] also highlighted the same issue as one of the factors affecting the SM behavior. Not taking the illness seriously by SM attempters can cause many problems, such as progression of the disease, occurrence of complications, need for long-term hospitalization, and increases in health services costs [17–19, 26]. In the same line, the meta-analysis conducted by Azami-Aghdash et al. [3] indicated that mild symptoms accounted for the largest impact (63.7%) among the effective factors in SM. Thus, it is necessary to provide opportunities for education in this respect in order to reduce the conditions leading to SM.

The deep mistrust in the medical profession and the rare honesty among physicians was another underlying factor in SM. These results were consistent with those of the studies by Mortazavi et al. [27], Azami-Aghdash et al. [3], and Jafari et al. [22]. Generally, the process of building trust bears on treatment acceptability among patients. This necessitates paying special attention by healthcare policymakers with the aim of eliminating the factors resulting in the above-mentioned mistrust through the development of public knowledge about physicians’ duties and treatment processes.

Moreover, individual and social problems reportedly encouraged SM. According to the participants, socioeconomic problems stimulated SM. The findings of the studies performed by Azami-Aghdash et al. [3], Beza et al. [12], Mortazavi et al. [27], and Wen et al. [10] also revealed that SM practitioners avoided consulting physicians due to their socioeconomic problems, such as financial and time constraints. Similarly, Jafari et al. [22] found that 82 and 45.5% of the participants practiced SM due to time and cost savings, respectively. Hence, healthcare policymakers and administrators have to adopt appropriate measures in order to improve the underlying conditions facilitating SM and to reduce patients’ waiting times and financial burdens in medical centers.

The main concern among SM practitioners was to deal with their illnesses/symptoms both simply and quickly. The study results showed that the people tended towards SM so as to cope with their health problems as simply and quickly as possible. Consistently, Wen et al. [10], Le et al. [11], and Mortazavi et al. [27] mentioned simplicity and time-efficiency as two major factors that encouraged SM and discouraged medical treatment. Furthermore, Albusalih et al. [14] stated that the most common reasons for SM were mild problems and previous experience with medicine. The outlined concern requires attraction of public’s attention to the consequences and complications of SM, so that people would choose whether or not to practice SM in an informed manner.

The present study findings showed that people employed different methods to decide on SM. According to their desire to seek for rapid relief from illnesses/symptoms, the participants had self-reportedly employed different methods, such as searching the Internet and sharing their SM experiences. The practitioners’ efforts to address their main concern were summarized as “to avoid being trapped in the vortex of illness”. Other research findings have also indicated few agents for SM, including family members [12, 28], friends and neighbors [13, 28], peers [10], and non-medical staff like pharmacists [2, 28]. These findings were consistent with those of the present study, thus emphasizing the influence of others on the decision to practice SM. The meta-analysis performed by Azami-Aghdash et al. [3] also revealed a 35.9% share for unprofessional advice in SM cases. Jafari et al. [22], too, showed that 64.6% of the patients acted according to such advice (friends, neighbors, and family members). In the same line, the findings of the studies by Le et al. [11], Loyola Filho et al. [13], Mortazavi et al. [27], and Wen et al. [10] demonstrated that having suffered from similar illnesses in the past and having a history of using similar medications facilitated the decision on SM. According to Jafari et al. [22] and Azami-Aghdash et al. [3], the previous experience with the same illness and the same medications accounted for 73 and 51.4% of the cases of SM, respectively. The strategies for inhibiting the growing phenomenon of SM may include changing the public attitudes towards SM and raising awareness about the different conditions facing different patients.

The implications of SM were manifested through a range of satisfaction levels. Most of the participants mentioned a temporary sense of satisfaction as the result of SM with few components, including time-efficiency, cost-effectiveness, and transient relief. Consistently, Beza et al. [12], Wen et al. [10], and Jafari et al. [22] reported that the cost-effectiveness of SM was a strong promoter compared to the financial burdens imposed by physicians and medical procedures. The cost-effectiveness reportedly embraces both time and cost savings, which are possible to achieve by avoiding physicians or medical centers [29, 30]. Since the resultant satisfaction is often temporary, it is necessary to raise awareness about the consequences of avoiding physicians and medical treatments so that people would make a well-informed decision. Quite apart from this temporary sense of satisfaction, SM led nowhere but to long-term dissatisfaction in some cases where the high cost of delayed medical procedures and prolonged treatments by SM were self-reportedly found to be the components of dissatisfaction.

The present study population included a group of Iranian people with a history of SM in two southern provinces. Despite the detailed data analysis and the careful development of categories from the process of SM, it requires a cautious approach to generalize the study results to other populations. A major advantage of this study was interviewing general practitioners, specialists, and pharmacists through theoretical sampling to complete the process and relate the categories as perfectly as possible.

## Conclusions

This was the first qualitative study with a GT-based approach to SM in Iran. The study provided valuable information for healthcare policymakers to identify the components affecting the process of SM for effective planning to address this pervasive health problem. The study results, in the form of a conceptual model, indicated that the process of SM included a series of underlying factors, facilitating factors, strategies, and consequences of importance to be considered by health policymakers. Some recommended solutions to this problem are to broaden the public knowledge and attitude in this regard, to provide more healthcare services tailored to the population, to deliver comprehensive healthcare consulting services to develop further informed decisions and choices, and finally to focus on the improvement of patients’ trust and patient-physician relationships.

## Supplementary information

**Additional file 1.** Interview question guide for interviews

## Data Availability

The data analyzed in the current study are available from the corresponding author upon reasonable request.

## Abbreviations

GT: Grounded theory
SM: Self-medication
