# Supplementary material for: A model to explain self-medication by Iranian people: a qualitative grounded theory study
Source: BMC Public Health. 2019 Dec 2;19:1609. doi: 10.1186/s12889-019-7953-0 (PMC6889693; doi:10.1186/s12889-019-7953-0)
Supplement: Supplementary file 1 — Additional file 1. Interview question guide for interviews [file 12889_2019_7953_MOESM1_ESM.docx]

**Appendix 1. Interview question guide for interviews**

1. *How do you describe your self-medication experience?*
2. *Could you please explain more about your decision?*
3. *Could you please mention the basis of your decision for self-medication?*
4. *How do you think about facilitators of self-medication in your decision?*
5. *How do you think about barriers of self-medication in your decision?*
6. *What were effective factors in your decision?*
7. *How do you use strategies in the process of self-medication?*
